# Inhibition of nuclear factor kappa-B signaling reduces growth in medulloblastoma *in vivo*

**DOI:** 10.1186/1471-2407-11-136

**Published:** 2011-04-14

**Authors:** Susan E Spiller, Naomi J Logsdon, Lindsey A Deckard, Harald Sontheimer

**Affiliations:** 1 Department of Pediatrics, University of Alabama at Birmingham, 1600 7th Ave S., Birmingham, AL 35294, USA; 2 Civitan International Research Center 1719 6thAve. S., Birmingham, AL 35294, USA; 3 Department of Neurobiology, University of Alabama at Birmingham, 911 Shelby Biomedical Research Building, 1825 University Blvd, Birmingham, AL 35294, USA

## Abstract

**Background:**

Medulloblastoma is a highly malignant pediatric brain tumor that requires surgery, whole brain and spine irradiation, and intense chemotherapy for treatment. A more sophisticated understanding of the pathophysiology of medulloblastoma is needed to successfully reduce the intensity of treatment and improve outcomes. Nuclear factor kappa-B (NFκB) is a signaling pathway that controls transcriptional activation of genes important for tight regulation of many cellular processes and is aberrantly expressed in many types of cancer.

**Methods:**

To test the importance of NFκB to medulloblastoma cell growth, the effects of multiple drugs that inhibit NFκB, pyrrolidine dithiocarbamate, diethyldithiocarbamate, sulfasalazine, curcumin and bortezomib, were studied in medulloblastoma cell lines compared to a malignant glioma cell line and normal neurons. Expression of endogenous NFκB was investigated in cultured cells, xenograft flank tumors, and primary human tumor samples. A dominant negative construct for the endogenous inhibitor of NFκB, IκB, was prepared from medulloblastoma cell lines and flank tumors were established to allow specific pathway inhibition.

**Results:**

We report high constitutive activity of the canonical NFκB pathway, as seen by Western analysis of the NFκB subunit p65, in medulloblastoma tumors compared to normal brain. The p65 subunit of NFκB is extremely highly expressed in xenograft tumors from human medulloblastoma cell lines; though, conversely, the same cells in culture have minimal expression without specific stimulation. We demonstrate that pharmacological inhibition of NFκB in cell lines halts proliferation and leads to apoptosis. We show by immunohistochemical stain that phosphorylated p65 is found in the majority of primary tumor cells examined. Finally, expression of a dominant negative form of the endogenous inhibitor of NFκB, dnIκB, resulted in poor xenograft tumor growth, with average tumor volumes 40% smaller than controls.

**Conclusions:**

These data collectively demonstrate that NFκB signaling is important for medulloblastoma tumor growth, and that inhibition can reduce tumor size and viability *in vivo*. We discuss the implications of NFκB signaling on the approach to managing patients with medulloblastoma in order to improve clinical outcomes.

## Background

Medulloblastoma is largely a cancer of children, with 75-80% of cases diagnosed in individuals younger than fifteen years; some are diagnosed in infancy [1-3]. It is a very aggressive and invasive cancer which spreads primarily via cerebral spinal fluid to metastasize anywhere in the leptomeninges, or, in advanced disease, hematogenously to invade any body part. It is suspected to arise from cerebellar granule cell precursors [1,4] based on its primitive neuronal histology and location in the midline posterior fossa. Survival is achievable in many children, dependent on a number of factors, yet recurrence holds a dismal prognosis [3]. Current understanding of the biology of medulloblastoma cannot fully provide an explanation for medulloblastoma occurrence, proliferative properties, migratory activity, or chemotherapy resistance.

Prognosis has improved over the last half century with the addition of radiation therapy and chemotherapy. In spite of these advances, there remains a considerable unmet need to increase survival rates, especially in high-risk disease. Further, targeted therapies need to be identified such that normal developing brain tissue will be spared, thereby avoiding the disabling sequelae which are, unfortunately, commonplace in survivors. These goals are more likely to be achieved through better understanding of the biology of the disease and exploiting features unique to the tumor rather than by the current strategy of damaging tumor cells more than normal cells.

Despite comprehensive studies to identify risk factors associated with medulloblastoma, no environmental risk factor has been linked to development of medulloblastoma. A variety of chromosomal abnormalities have been reported, and defects in signaling pathways such as Wingless (Wnt) and sonic hedgehog (SHH) have been identified in some sporadic and heritable forms of medulloblastoma [1,3,5], but these represent a minority of cases.

Current research pertaining to cancer and immune response has revealed an association between nuclear factor kappa B (NFκB) signaling and tumorigenesis. Over the past 25 years, NFκB has been described and characterized through a wide range of normal and pathologic model systems [6]. NFκB is a family of transcription factors that regulate genes involved in cell growth, apoptotic cell death, adhesion and angiogenesis. Although only the related viral oncogene v-rel is acutely transforming, growing evidence implicates nearly all members of the NFκB family in human malignancy [7,8]. Chromosomal abnormalities within the genes of these transcription factors are found in many solid and hematopoietic tumors. Also, many cancers have mutations affecting the activity of upstream regulators [6]. Moreover, many forms of leukemia and a wide variety of solid tumors demonstrate constitutive activation of this otherwise tightly regulated pathway [9] by increasing pathway stimulation or by inactivating negative feedback molecules [9,10]. Some of the most aggressive malignancies of childhood, including neuroblastoma, rhabdomyosarcoma, Wilms tumor, and retinoblastoma have also been reported to involve NFκB.

NFκB is normally quiescent in cells and only becomes activated in response to stress signals such as pro-inflammatory cytokines. In the canonical NFκB pathway, a heterodimer consisting of two subunits, p65 (RelA) and p50, is sequestered in the cytoplasm by an inhibitory protein, IκB-α, under normal physiological conditions. Upon induction of the pathway, the inhibitor IκB-α becomes phosphorylated by an IκB kinase (IKK), which leads to ubiquitination of IκB. The inhibitor is thus targeted to the ubiquitin-proteasome for degradation, and the NFκB heterodimer p65/p50 is free to translocate to the nucleus and begin altering gene expression [10,11]. Some specific downstream NFκB targets include survival genes Bcl-xl and XIAP; adhesion molecules ICAM-1, VCAM-1 and ELAM-1; metastasis promoting gene MMP-9; angiogenesis factors VEGF and TNFα; proliferation genes cyclin D1 and C-myc; and a host of other genes known for their association with proliferation, inflammation, and immortalization of cells [6,10,12].

Because of the prominence NFκB signaling has gained in adult cancer research and evidence of its activity in some high grade pediatric malignancies, we have asked whether this pathway could be a dominant feature of medulloblastoma. Our studies have established that p65 is indeed over expressed in primary tumor samples and in tumor cell lines. Furthermore, multiple drugs that inhibit NFκB cause apoptotic cell death in all four cell lines tested. Finally, disruption of NFκB signaling using a dominant negative variant of the endogenous inhibitor of NFκB, dnIκB, resulted in reduced xenograft tumor growth. We have, therefore, established that NFκB plays a role in medulloblastoma and that it may be a target for therapeutic intervention.

## Methods

### Chemicals

All chemicals were obtained from Sigma-Aldrich, St. Louis, MO unless otherwise indicated. Stocks were prepared as follows: curcumin, 10 mM in ethanol; bortezomib (LC laboratories, Woburn, MA), 200 mg/mL in DMSO, then diluted to 10 mM in phosphate buffered saline (PBS) pH 7.4; pyrrolidine dithiocarbamate ammonium salt (PDTC), 10 mM in PBS; diethyldithiocarbamate sodium salt (DDTC), 10 mM in PBS; sulfasalazine (SAS) 10 mM in 0.1 M saline, pH7.4; doxycycline, 10 μg/mL in sterile water; TNFα (R&D Systems, Minneapolis, MD), 100 μg/mL in sterile PBS+0.1% bovine serum albumin.

### Cell Culture

Experiments using cultured medulloblastoma cells were performed on two commercially available cell lines, Daoy and D283, (American Type Culture Collection, Manassas, VA) and two cell lines, D425 and D458 [13,14], established from primary medulloblastomas (kindly provided by Dr. D. Bigner, Duke University, Durham, NC). Cell cultures were maintained in MEMα supplemented with 2 mM _L_-glutamine (Mediatech, Manassas, VA) and 10% characterized fetal bovine serum (FBS) (HyClone, Logan, UT), or in Richter's improved MEM Zinc Option containing 10 mM HEPES and 0.22% sodium bicarbonate (Life Technologies), 2 mM _L_-glutamine, and 10% FBS. U-87MG Grade III glioma cell line (kindly provided by Dr. D. Bigner, Duke University, Durham, NC) was grown in Dulbecco's Modification of Eagle's Medium/F-12 supplemented with 7% FBS and 2 mM _L_-glutamine (Mediatech). Mouse neurospheres were derived from embryonic day 14 mouse cortex (StemCell Technologies Inc, British Columbia, Canada) and were maintained in 90% NeuroCult NSC Basal Medium with 10% NeuroCult NSC Proliferation Supplements (StemCell Technologies); 20 ng/mL rhEGF was added just before use. All medulloblastoma cells and neurospheres were maintained at 37°C in a 95% O_2_-5% CO_2 _humidified atmosphere; U-87MG cells were incubated in 90%O_2_-10%CO_2_.

### Proliferation assays

Cells were seeded in 24-well dishes at densities that had been determined to allow for exponential growth for the duration of the experiment. For the time course, cells were left untreated or were treated for 3 days at the IC90 for each line (325 nM PDTC for Daoy and 350 nM for D425). Cells were counted using a Beckman Multisizer 3 Coulter counter. For dose-response experiments, cells were grown for 3-4 days in the absence or presence of varying concentrations of each drug and counted as above.

### Annexin-V staining

Apoptosis was measured with annexin V-Cy3 staining, following manufacturer's instructions (BioVision Inc, Mountain View, CA). At least 100 cells were counted for each experiment. Positively staining cells in each low power field were identified by fluorescence microscopy. The number of positive cells was divided by the total number of cells in those fields and reported as % positive for annexin V.

### Tissue

Human autopsy tissue was obtained from the University of Alabama at Birmingham (UAB) Tissue Collection and Banking Facility, Cooperative Human Tissue Network Southern Division. Smo/Smo transgenic medulloblastoma mice with constitutive expression of *Smoothened *in cerebellar granule neurons [4] were generously provided by Dr. Jim Olson, Fred Hutchinson Cancer Research Center, Seattle, WA. All studies were performed in accordance with standards of the UAB Institutional Review Board (IRBN080731009, X070829010).

### Whole cell or total tissue lysates

Cell pellets collected from 10 cm culture dishes were rinsed in PBS and lysed in RIPA (50 mM Tris-HCl pH 8.0, 150 mM NaCl, 1% IGEPAL detergent, 0.1% SDS, 0.5% Na^+ ^deoxycholate) containing 1X final concentration protease and phosphatase inhibitors (Sigma-Aldrich). Tissues (10-30 mg) were homogenized on ice in lysis buffer (100 mM Tris-HCl pH 7.4, 1% SDS) containing protease and phosphatase inhibitors. Samples were sonicated briefly, incubated at 4°C with occasional agitation for 1 hour, then clarified by centrifugation at 14,000 X g for 30 minutes at 4°C. Proteins were quantified using the modified detergent-compatible Lowry assay (BioRad Laboratories, Hercules, CA).

### Nuclear and Cytoplasmic extracts

Frozen tissue was homogenized in cavitation buffer (5 mM HEPES, pH 7.4; 3 mM MgCl_2_; 1 mM EGTA; 250 mM sucrose) containing protease and phosphatase inhibitor cocktails (Sigma-Aldrich) and 0.1 μM okadaic acid (Alexis Biochemicals, San Diego, CA). Cell lysis was achieved by nitrogen cavitation (250 psi, 5 minutes on ice). The resulting lysate was centrifuged at 700 relative centrifugal force (rcf) for 10 minutes. Cytosolic fractions were obtained by centrifuging the low-speed supernatant at 16,000 rcf for one hour. Nuclei were purified from the low-speed pellet by washing twice in cavitation buffer at 2700 rcf for 5 minutes each. The washes were repeated in cavitation buffer containing 0.5% IGEPAL detergent (Sigma-Aldrich), and then the pellet was resuspended and loaded onto a 1 M continuous sucrose gradient prior to centrifugation at 4°C, 2700 rcf for 10 minutes. The pellet was recovered and the sucrose gradient repeated, followed by a wash in cavitation buffer containing detergent for 5 minutes at 2700 rcf, and then a final wash in cavitation buffer without detergent for 5 minutes at 16,000 rcf. Extracts were obtained from the purified nuclei by resuspending in lysis buffer (10 mM Tris pH 7.4, 150 mM NaCl, 1mM EGTA, 1 mM EDTA, 1 mM IGEPAL detergent) containing protease and phosphatase inhibitors (Sigma-Aldrich) and agitating at 4°C for 30 minutes. Nuclear extracts were clarified by centrifugation at 16,000 rcf for 30 minutes at 4°C.

Nuclear and cytoplasmic extracts of cultured cells or frozen tissue were also obtained using the Pierce NE-PER kit according to manufacturer's instructions (Thermo Scientific Pierce Protein Research Products, Rockford, IL). Cells were either untreated, or were treated for 30 minutes with 15 ng/mL TNFα. Proteins were quantified as above.

### Western Blots

Fifteen to 20 micrograms total protein per lane were separated on 10%, 12%, or 15% SDS PAGE gels and transferred to PVDF membranes (Millipore, Billerica, MA). Membranes were blocked in TBS-T (20 mM Tris-HCl pH 7.6, 137 mM NaCl, 0.1% Tween-20) containing 5% nonfat dry milk for 1 hour at room temperature. Primary antibodies were applied overnight at 4°C in TBS-T+5% milk. Primary antibodies included: NFκB subunit p65, (Santa Cruz Biotechnology, Inc, Santa Cruz, CA); phosphorylated (S536 or S276) NFκB subunit p65, (Cell Signaling Technology, Danvers, MA); IκB-α (Santa Cruz); tubulin (Abcam Inc, Cambridge, MA); histones (Chemicon/Millipore) (MAb 052); actin (Sigma-Aldrich); cleaved caspase 3 (Cell Signaling). After extensive washing, membranes were incubated with the appropriate secondary antibody (goat anti-mouse or goat anti-rabbit-HRP conjugate, Santa Cruz) at room temperature for one hour. Detection was accomplished with ECL plus detection reagent (GE Healthcare Biosciences, Piscataway, NJ) and images were collected on a Kodak Image Station 4000MM. Membranes were stripped for 1 hour at 50°C in strip buffer (62.5 mM Tris-HCl pH 6.7, 2% SDS, 100 mM β-mercaptoethanol), rinsed extensively in TBS-T, and re-probed as needed.

### Immunohistochemistry

Slides of human primary medulloblastoma were obtained from the Cooperative Human Tissue Network Pediatric Division (Columbus Children's Hospital, Columbus, OH). The sections were deparaffinized in Citrisolv (Fisher Scientific, Pittsburgh, PA) and a standard citrate buffer antigen retrieval technique [15] was used to unmask protein epitopes. After blocking, the sections were incubated in phos(S276)p65 primary antibody (Cell Signaling, Boston, MA) overnight at °C. After washing, sections were incubated in an HRP-conjugated goat anti-rabbit secondary antibody at room temperature for 1 hour. Biotin tyramide (NEN Life Science Products, Perkin Elmer, Waltham, MA) signal amplification was used to enhance detection with the avidin biotin complex (ABC) method (Vector Laboratories, Burlingame, CA) and diaminobenzidine (Sigma-Aldrich) chromagen substrate. The sections were counterstained with hematoxylin. These studies were performed at the UAB Neuroscience Molecular Detection Core, supported by P30 NS47466.

### DNA construct

A dominant negative form of IκB-α (S32A and S36A) was amplified from the plasmid pCMV-dnIκBαM (Clontech, Mountain View, CA) and cloned into the pTRE-Tight-Bi-AcGFP1 vector (Clontech) at the KpnI and NotI sites to create pTRE-Tight-Bi-AcGFP-dnIκB (Additional File 1 A). This vector co-expresses AcGFP with dnIκB from a bidirectional promoter upon activation of the tet response element. Sequences were confirmed by automated sequencing.

### Double-stable cell line 4H10

D425 cells were first transfected with the pTet-off plasmid (Clontech) to create a tet-responsive line expressing the tTA protein. Cells were transfected with Lipofectamine at a 1:5 DNA to Lipofectamine ratio according to the manufacturer's instructions (Invitrogen, Carlsbad, CA). The cells were incubated overnight with the Lipofectamine/DNA complexes, and the next day cells were washed to remove the DNA. Twenty four hours later cells were re-plated in a 6-well dish for selection with 0.4 mg/mL geneticin (Invitrogen). After 10 days, cells were expanded to a 6 cm dish, and a week later colonies were isolated using cloning disks (RPI) and expanded for testing. Clones were evaluated for expression of tTA using a luciferase assay (see below). Selection was maintained at 0.1 mg/mL geneticin.

The double stable cell line expressing dnIκB and AcGFP under control of the tet response element was created by transfecting the D425 tet off cell line with the pTRE-Tight-Bi-AcGFP-dnIκB construct and a linear hygromycin marker (Clontech) in a 10:1 ratio as above. Clones were selected by limiting dilution in 96-well dishes using 0.2 mg/mL hygromycin B (EMD Biosciences, Gibbstown, NJ). Doxycycline (DOX) (Sigma-Aldrich) was included at 100 ng-1 μg/mL to repress expression of dnIκB. Clones were expanded and tested by FACS (UAB CFAR Flow Cytometry Core) for expression of AcGFP by washing out DOX as follows. Cells were harvested, washed twice in PBS, and re-plated in fresh media containing no DOX. Three to 6 hours later the media was replaced again. The clone displaying the highest fluorescence, 4H10, was further evaluated; selection was maintained at 0.1 mg/mL hygromycin.

### Luciferase assays

Tet off clonal cell lines were evaluated for expression of tTA by transiently transfecting with the reporter plasmid pTRE-HA-Luc (Clontech) using Lipofectamine as above. The next day cells were washed and split into 2 identical wells, and DOX was added to one well at 2 μg/mL. The following day, cells were washed and incubated with beetle luciferin (Promega, Madison, WI) and luminescence was measured on a LumiStar luminometer (BMG Labtech, Japan). Cells were then counted and luminescence was normalized to cell number. Fold induction was calculated as relative luciferase units (RLU): RLU (No DOX)/RLU (DOX). A cell line with greater than 20 fold induction and little to no background was selected for further work.

Double stable cell lines expressing dnIκB under control of the tet response element were evaluated for biological response by transiently transfecting with the reporter plasmid p5X NFκB-Luc using Lipofectamine as described. The next day, DNA-containing medium was removed and cells were cultured in 100 ng/mL DOX or washed as described above to remove DOX. Two days later, cells were collected and assayed for luciferase activity as above.

### Xenografts in athymic mice

Twenty-four 6-8 week old female athymic mice were randomized to two arms, DOX or No DOX. Mice in the DOX arm received mouse pellets containing 200 mg/kg [16] DOX (Bio-Serv, Frenchtown, NJ) for 1 week prior to tumor implantation. *4H10 cells *(D425 medulloblastoma cells containing dnIκB construct) were cultured in 100 ng/mL DOX until 24 hours prior to injection of flank tumors. DOX was washed out of half the cells as described above for injection into mice in the No DOX arm. One million cells were resuspended in 200 μL of a solution containing 50% serum-free media and 50% Matrigel (BD Biosciences, San Jose, CA) and injected subcutaneously into the right flank of the mice, one injection per animal, on Day 1. Tumors were measured with digital calipers and tumor volume was calculated using the formula A^2^xB/2, where A is the short diameter of the tumor, and B is the long diameter perpendicular to A. Mice in all studies were weighed and assessed for general health once a week, and tumors were measured twice a week. Animal experiments were performed according to the National Institutes of Health Guide for the Care and Use of Experimental Animals and approved by the Institutional Animal Care and Use Committee (IACUC APN 08607). Animals were sacrificed according to humane guidelines provided by the IACUC. The study endpoint was Day 37, when several tumor volumes were estimated at nearly 10% of the animals' weight. Tumors were removed and measured in three dimensions, and weighed to validate the accuracy of the *in situ *measurements.

### Statistics

The xenograft study used one tail Student's t-test assuming equal variance. In figures, all error bars represent standard error of the mean (SEM).

## Results

### Medulloblastoma cells in culture are exquisitely sensitive to pharmacological inhibitors of NFκB

**Figure 1:**
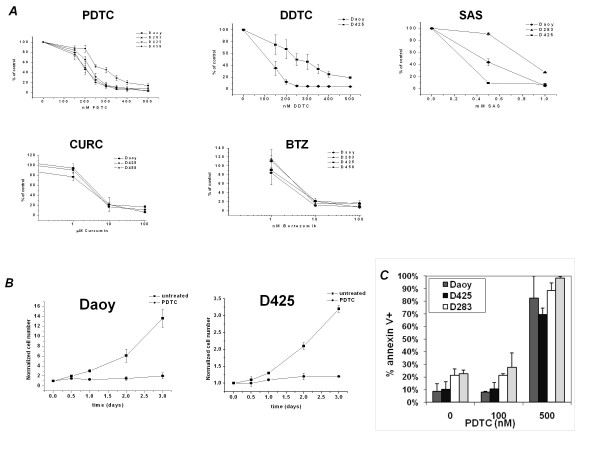
**Pharmacological inhibition of NFκB in medulloblastoma cell lines**. A: Dose Dependent Survival: Dose response of medulloblastoma cell lines (cell number normalized to control) to NFκB inhibitors: pyrrolidine dithiocarbamate = PDTC, diethyldithiocarbamate = DDTC, sulfasalazine = SAS, curcumin = CURC, bortezomib = BTZ. (n = 3) Drug applied on day 0, cell count day 3 B: Time dependent survival: Time course of Daoy and D425 cells in response to IC90 values of PDTC (cell number normalized to control). Daoy 325 nM PDTC; D425 350 nM PDTC. (n = 3) Drug was applied only once on Day 0 C: Apoptosis: Annexin V staining for apoptosis in cell lines after 3 days in culture with designated concentrations of PDTC (n = 3 or more)

Several pharmacological inhibitors of the NFκB pathway were tested on multiple human medulloblastoma cell lines (Figure 1A). After three days, less than 20% of cells survived exposure to nanomolar concentrations of pyrrolidine dithiocarbamate (PDTC), diethyldithiocarbamate (DDTC), and bortezomib (BTZ). Cells were also sensitive to micromolar concentrations of curcumin (CURC) and sulfasalazine (SAS). This inhibition of cell growth was dose dependent and persistent over time: using PDTC at the IC90, Daoy and D425 cell lines failed to proliferate after three days following a single administration of drug at day zero (Figure 1B).

To determine the nature of PDTC-induced inhibition of cell growth, cells were assayed for apoptosis. Cells were treated with PDTC at varying concentrations for 3 days and then stained with annexin V, a protein that binds to cell surface phosphatidylserine, which is expressed when cells undergo apoptotic cell death. Staining demonstrated that the cause of decreased cell number seen in dose response curves is largely due to apoptosis (Figure 1C). This was true for all medulloblastoma cell lines tested.

**Figure 2:**
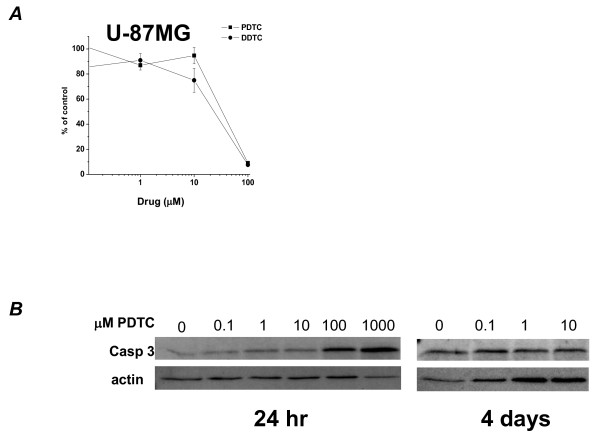
**Pharmacological inhibition of NFκB in other cells**. A: DDTC, PDTC in glioma: Dose response of human U-87MG glioma cells to PDTC and DDTC after 4 days in culture (cell number normalized to control). n = 3 B: PDTC in immature neurons: Dose response of mouse neurosphere stem cells to PDTC (mM): Western analysis probing with anti-cleaved caspase 3(Casp 3) (band ~19kD).

Interestingly, U-87MG glioma cells treated with DDTC and PDTC were insensitive to both drugs at concentrations <1,000 nM (Figure 2A). Only concentrations >10 μM inhibited U87 proliferation. Thus, medulloblastoma cells are far more sensitive to NFκB inhibition than another malignant brain tumor cell line. There are reports of NFκB constitutive activity in glioma [17,18] and reports to the contrary [19,20]. U-87MG cells are, however, very sensitive to sulfasalazine [20], due to a mechanism of action separate from its NFκB inhibition (discussed below).

Because NFκB is an important signaling pathway in many homeostatic functions of normal cells, we tested the effect of an NFκB blocker on normal neural stem cells. Neurospheres acquired from mouse E14 cortical neural stem cells were treated with a broad range of PDTC concentrations and evaluated at 24 hours and at 4 days post-treatment. Because these cells grow in spheres comprising hundreds of cells each, an accurate cell count was not achieved. However, visual inspection of spheres shows no morphologically apparent difference in sphere size, number or integrity at doses of 0.1 μM to 10 μM. At 100 μM PDTC, a dose that would be quite toxic to medulloblastoma cells, subtle changes were detected: there were fewer large spheres, and smaller spheres were less aggregated. At 1mM, all spheres were smaller and less smooth, suggesting disrupted adhesion integrity, and free floating cells were more numerous with a crenated appearance, suggesting cell death (Additional File 1 B). Western blot analysis of neurosphere lysates reveals increased cleaved caspase 3 expression after 24 hours of 1 μM PDTC treatment and a large amount of cleaved caspase 3 expression from cells treated with >100 μM PDTC. At 4 days, the low concentration of PDTC (0.5-1 μM) needed to eliminate most medulloblastoma cells did not have much effect on growth and survival of nonmalignant mouse neural stem cells. There was not a substantial increase in cleaved caspase 3 expression among cells treated with 0-10 μM PDTC (Figure 2B). Taken together, the data indicate that medulloblastoma cells are exquisitely sensitive to NFκB blockade and will not proliferate when exposed to NFκB inhibitors at concentrations that do not affect normal neural stem cells or other malignant cell lines.

### Nuclear p65 protein expression is inducible but not constitutive at high levels in cultured medulloblastoma cells

**Figure 3:**
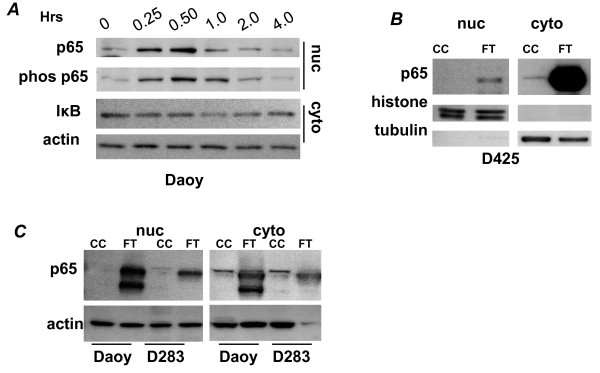
**NFκB expression in medulloblastoma *in vitro *and *in vivo***. A: Activation of NFκB: Timecourse of NFκB activation by 15 ng/mL TNFα in Daoy cells by probing for nuclear p65, nuclear phos(S276) p65, & cytoplasmic IκB. nuc = nuclear fraction, cyto = cytoplasmic fraction. p65 band ~70kD; phos p65 band ~97kD; IκB band ~37kD B: Tissue Expression: expression of NFκB p65 subunit in D425 cells grown in culture (CC) compared to D425 cells grown in a flank tumor model (FT). Nuclear (nuc) and cytoplasmic (cyto) fractions were separated by differential centrifugation following lysis by nitrogen cavitation as described. C: p65 expression by Western in cultured cells vs flank tumors for 2 additional cell lines. CC = cells in culture, FT = flank tumor. p65 band~60kD. Extracts were obtained using the NE-PER kit as described.

The sensitivity of medulloblastoma cells to inhibitors suggests that NFκB is an important and probably aberrantly regulated pathway in these cell lines and tumors. In cultured cells, however, significant p65 protein expression in the nucleus was only apparent with stimulation by tumor necrosis factor alpha (TNFα). Maximal response is seen at 30 minutes after application of TNFα. p65 expression returns to baseline by four hours. IκB expression in the cytoplasm decreased with TNFα stimulation, as expected [11] (Figure 3A). These results suggest that p65 is not highly constitutively active in cultured cells without application of external stress, though a low level of baseline expression is evident. The reason these cells are so sensitive to NFκB blockade is unclear but may be due to a reliance on low level function which is sensitivity to any perturbations in the pathway. It is possible these changes are too subtle to detect by Western blotting.

### Xenograft flank tumors from medulloblastoma cell lines over express both nuclear and cytoplasmic p65

Because p65 protein is inducible with TNFα stimulation in cultured cells, but its activity was difficult to detect in resting cells, we reasoned that NFκB signaling in tissue may be dependent on factors not available in culture. We prepared tumors from cell lines to test this idea. D425 cells were used to establish flank tumors in athymic mice. Nuclear extracts from tumors show enhanced nuclear p65 by Western compared to the same cell lines in culture. The cytoplasmic p65 is extremely abundant when D425 cells are grown in mice as flank tumors, indicating that characteristics of the tumor environment stimulate robust NFκB signaling. Figure 3B shows Western data from one representative tumor; 3 additional tumors and 3 more passages of D425 cells were tested with similar results. Daoy and D283 cell lines were also used to develop flank tumors and demonstrated the same dramatic upregulation of p65 *in vivo *(Figure 3C).

### NFκB is over expressed in human and mouse medulloblastoma tumors

**Figure 4:**
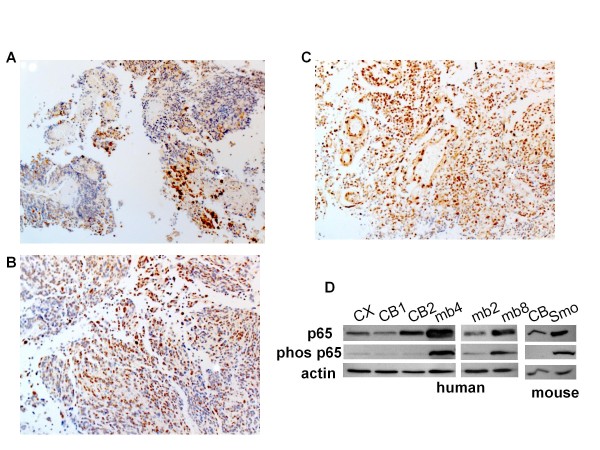
**Primary tumor expression of NFκB**. A-C: Phosphorylated p65 in primary medulloblastoma: immunohistochemical staining of three different primary medulloblastoma tumors with phos(S276)p65 antibody. Magnification: A = 10X; B = 20X; C = 10X. C shows endothelial cell positivity in addition to tumor cells. D: Expression of p65 and phos (S536) p65 in normal brain and tumor in humans [CX = cerebral cortex, CB1 and CB2 = cerebella from 2 different autopsy specimens; mb4, mb2, mb8 = surgical samples of 3 different patient medulloblastoma tumors] along with normal C57Bl/6 mouse cerebellum (CB) compared to Smo/Smo mouse (Smo) cerebellar tumor.

Primary tumor samples from three cases of medulloblastoma were obtained, and staining with hematoxylin and eosin (H&E) was used to identify the location of tumor cells (not shown). Immunohistochemical (IHC) staining showed that at least 50-90% of evaluable tumor cells strongly express phos(S276) p65, the nuclear transcriptionally active form of p65 (Figure 4A-C). To corroborate this IHC finding, total tissue lysates from 3 more human primary medulloblastoma tumor samples were probed by Western blotting for total p65 and phos(S276)p65. Controls for this assay are human cerebral cortex and cerebellum from adult autopsy tissue. These human tumor samples showed higher levels of total and phosphorylated p65 compared to non-malignant tissue. Additionally, tissue from a brain tumor that developed in a Smo/Smo transgenic medulloblastoma mouse [4] showed an overabundance of p65 and phos(S276) p65 compared to a wild type C57Bl/6 mouse cerebellum (Figure 4D).

### NFκB signaling is important in normal growth control of medulloblastoma cells *in vitro *and *in vivo*

Medulloblastoma cells in culture grow poorly and undergo apoptotic cell death in response to drugs that inhibit the NFκB pathway. However, no drug tested is specific for NFκB alone. To more specifically determine the necessity of normal NFκB signaling for survival and proliferation of medulloblastoma, we genetically manipulated D425 cells to prevent pathway activity. A dominant negative form of IκB (dnIκB) was constructed. The dominant negative protein has two mutations that eliminate phosphorylation sites, preventing IκB from being phosphorylated. The resulting protein cannot be degraded, so it can create a chronic blockade of p65 nuclear translocation, thus blocking NFκB -driven transcription. The dnIκB construct was prepared in the inducible "Tet-off" system, which inhibits expression of the construct in the presence of doxycycline (DOX), but which induces full expression once the DOX is removed (No DOX). Inducing the construct by removing DOX was done to ensure that any effects were due to the construct alone and not to DOX.

**Figure 5:**
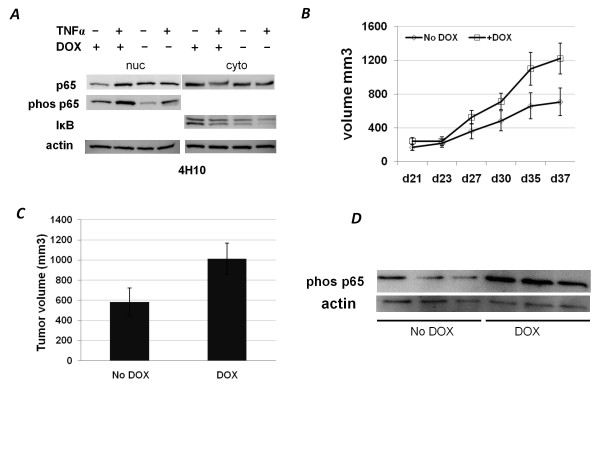
**Dominant negative inhibitor**. A. Induction of NFκB in 4H10 cells with expression of dnIκB (-DOX) or without dnIκB (+DOX) by 15 ng/mL TNFα for 30 min. nuc = nuclear fraction, cyto = cytoplasmic fraction. B. Mean tumor growth with and without dnIκB: Tumor volumes measured in athymic mice beginning at 21 days after xenograft 4H10 cell implantation and ending at day 37. C: Xenograft tumors were removed from animals and measured in 3 dimensions. Volumes were calculated using 4/3 π ABC, the volume of an ellipsoid, where A = length, B = width, C = breadth. No DOX represents animals not given doxycycline, therefore expression of dnIκB was permitted, blocking NFκB activation. D. Western of total tissue lysates from 4H10 (dnIκB) tumors removed from xenograft animals. Three tumors from animals with a normal diet (No DOX) are compared to three tumors from animals receiving DOX, which shuts off expression of dnIκB.

A stable cell line containing the dnIκB construct, 4H10, shows expression of p65 and phos(S536)p65 that can be stimulated with TNFα in much the way unaltered cultured medulloblastoma cells do (Figure 5A). However, when the construct was induced by washing away DOX, less nuclear p65 (specifically the transcriptionally active phosphorylated form) is evident, and there is not a significant decrease in cytoplasmic p65 with TNFα stimulation. This is consistent with a block of nuclear translocation. We then tested the biological activity of the construct. A reporter plasmid, p5x NFκB -Luc, bearing five copies of the NFκB promoter upstream of a luciferase reporter gene, was transiently transfected, and cells were stimulated with TNFα. DOX cells had a 13-fold increase in reporter expression upon TNFα stimulation, compared to only a 6-fold increase in No DOX cells (Additional File 1 C).

One possible explanation for the remaining NFκB activity in the induced (No DOX) 4H10 cells is that the dominant negative could be toxic to the cells and perhaps they are able to escape its effects despite stable drug selection. The co-expressed AcGFP was detected in only ~50% of induced cells analyzed by FACS (data not shown), despite rigorous drug selection. Another possibility involves potential overlap via the noncanonical NFkB pathway [6], which has not been investigated in this model.

The 4H10 cells containing inducible dnIκB were used to create flank xenograft tumors in athymic mice. Cells were grown with DOX in the medium, and 24 hours before flank implantation, half the cells were washed free of DOX. Mice that were assigned to the DOX arm were injected with cells continually exposed to DOX and were fed a diet containing DOX. Mice assigned to the No DOX arm were injected with cells that had DOX removed from the medium, and mice were given regular food. There were no apparent differences in the general health or food intake between the groups. Two mice initially had small tumors that after 5 weeks were imperceptible, and both were on the No DOX arm. One mouse on the DOX arm had a very rapidly growing tumor and had to be euthanized at four weeks, so it was excluded from data analysis. One mouse in the DOX arm died unexpectedly early in the trial and was also excluded. By study end at 5 weeks, the No DOX group had an average tumor volume 40% smaller than the DOX average volume (p < 0.03) based on measurements in live animals (Figure 5B). Tumors were removed, weighed, and remeasured, this time in 3 dimensions, and results were the same (Figure 5C). Western analysis on tumor tissue lysates confirms that the DOX tumors had higher phos(S276)p65 expression than No DOX tumors (expressing dnIκB). A representative Western of three animals from each arm of the study is shown in Figure 5D. Westerns of tumors from all remaining animals were consistent with this finding.

## Discussion

Our results indicate that NFκB is over-active in medulloblastoma, the most common malignant brain tumor in children. This was demonstrated in all six primary tumors examined by either IHC or western, as well as in xenografts of three human medulloblastoma cell lines. Further, limiting NFκB activity using a variety of pharmacological agents that target different steps of the pathway has a negative effect on tumor cell line viability and growth. These results implicate NFκB signaling as a necessary component of medulloblastoma tumor maintenance and tumor progression. We did not investigate the role of NFκB in tumorigenesis.

The five pharmacologic NFκB inhibitors we tested have different targets for disrupting NFκB signaling. Curcumin acts on IKK [37,38], a signal-dependent kinase, to block phosphorylation of p65 and IκBα by generation of reactive oxygen species [39]. Sulfasalazine inhibits phosphorylation of IκBα by blocking IKK activity [40,41]. Another activity of sulfasalazine is inhibition of system Xc(-), an amino acid transporter essential for cystine uptake and survival of glioblastoma [20]. We ruled out system Xc(-) as the mechanism of action in medulloblastoma cell lines by treating with S-4-CPG, a specific system Xc(-) inhibitor, with no effect on cell survival (data not shown). Bortezomib inhibits the ubiquitin-proteasome, thereby preventing IκB degradation [32]. Dithiocarbamates are metal chelators, antioxidants, proteasome inhibitors (under certain conditions) [42,43], and potent NFκB inhibitors [44], functioning to block IκBα release from NFκB in the cytoplasm. PDTC causes oxidation of NFκB, thereby decreasing DNA binding [45]. None of these agents is specific to NFκB but strike different targets within the pathway. Because these agents have many activities, yet have NFκB inhibition in common, we conclude that the commonality of tumor cell sensitivity to all of them lies in the NFκB pathway [10].

Because of the lack of specific pharmacologic inhibitors, we investigated a genetic blockade of the canonical NFkB pathway. The inducible dnIkB construct reduced growth of cells in culture by only about 25% of parent D425 cells, on average, as did a second dominant negative cell line established in Daoy parent cells (data not shown). Furthermore, the dnIκB in D425 cell xenograft tumors had a modest impact on tumor growth but did not completely eliminate it in most animals. It is likely, given these results, that pharmacologic inhibitors are more effective because they target NFκB-dependent as well as NFkB-independent pathways.

The role of NFκB has been extensively studied in neurons. p65/p50 heterodimers have been isolated from synapses [21]; neurotransmitters are among the many activators of NFκB [22]. Despite their neuronal lineage, medulloblastoma cells do not fire action potentials [46], so the role neurotransmitters play in NFκB signaling in medulloblastoma is not known. NFκB is activated by Ca^2+ ^influx via L-type Ca^2+ ^channels, but medulloblastomas do not express these channels, at least *in vitro *[46]. Therefore, neuronal physiology has not been helpful in clarifying the role of NFκB in medulloblastoma. However, it is clear that NFκB is activated in neurons in a variety of pathologic brain conditions. Neurons become more vulnerable to injury when NFκB is inhibited [23-25]. Thus, NFκB may be one mechanism used by neurons to survive insult [23]. We demonstrate that medulloblastoma cells are much more sensitive to NFκB inhibitors than normal immature neurons (Figure 2B), which would suggest that medulloblastoma cells rely on downstream targets, such as antiapoptotic genes, when they are physiologically challenged. In contrast, neuroprotection has also been reported by blocking NFκB in cerebellar granule neurons [25]. Indeed, inhibition of NFκB activity has been shown to promote as well as protect against neurotoxicity [25,26]. NFκB plays a role in cerebellar development during the first 1-2 weeks of life in mice and rats, and probably the first several months in humans [27,28]. It is suspected that NFκB signaling is involved in early granule neuron migration [27]. More studies are needed to determine whether NFκB inhibition could prevent medulloblastoma cell migration or metastatic spread, especially because this is a feature of medulloblastoma that is not very well understood.

Constitutive NFκB activity has been reported in adult malignant brain tumors and a number of other solid tumors in adulthood. There has been a focus on evidence relating chronic inflammation to cancer in recent years [10,29], including the role that NFκB plays in each. More specifically, neural stem cell inflammation and dysregulation of NFκB signaling could be a source of tumorigenesis in the brain [29]. However, the link between inflammation and cancer will be much harder to make in pediatric cases, because most tumors present in children who have no history of illness and no clinical inflammation. In fact, H&E staining of the three primary tumors presented here showed no inflammatory cell infiltration (data not shown). Yet, NFκB still seems to be significant in pediatric cancers. Retinoblastoma, a cancer of primitive neuroectodermal cells of the retina that occurs in very young children, is dependent on NFκB activity for survival [30]. Neuroblastoma, a tumor of children arising from neural crest cells, displays a spectrum of outcomes from spontaneous regression to rapid metastatic spread resistant to the most aggressive therapeutic interventions. Some neuroblastoma cell lines show high NFκB activity with dramatic response to its inhibition [31,32]. Additionally, high NFκB levels were found in rhabdomyosarcoma and Ewing's family of tumors [33,34]. There are two reported investigations of NFκB in medulloblastoma. The first examined methionine stress in brain tumors, and saw that methionine deprivation resulted in NFκB activation in one medulloblastoma cell line, causing rapid cell death [35]. This is consistent with our finding that NFκB is active and can be upregulated in cell lines. Specifically, we showed a large increase in detectable p65 when cultured cells are implanted as xenografts, compared to the same cells in culture. The other report indicates that 2-methylestrodiol, an inhibitor of NFκB, is pro-apoptotic in three medulloblastoma cell lines [36]. This result is very consistent with our data using five inhibitors of NFκB in multiple cell lines.

The clinical relevance of associating NFκB activity with malignancy may be substantial. First, there are a number of US Food and Drug Administration-approved or experimental drugs available that act to inhibit the NFκB signaling pathway. There are other anti-apoptotic signals in play in solid tumors, such that inhibition of NFκB is unlikely to change the course of the tumor when used as the sole anticancer therapy. Yet one must keep in mind that multi-drug therapy has long been the approach to cancer care because of this very issue. Second, blocking NFκB generally enhances the responsiveness of tumors to traditional chemotherapy [47,48]. This, however, is also not straightforward. NFκB activity is necessary for paclitaxel and doxorubicin cytotoxicity [49,50], and some traditional chemotherapies activate NFκB signaling [51,52]. Conversely, apoptosis induced by irinotecan, daunorubicin and cisplatin therapies is reportedly enhanced by blocking NFκB [47,49,53]. Once again the complexity of NFκB -modulated apoptosis is revealed, highlighting the importance of the proper balance of NFκB activity in individual tumors treated with specific chemotherapeutic agents. Cisplatin is an important anti-medulloblastoma drug; studies need to be performed to evaluate the effect of NFκB blockade on the chemosensitivity of medulloblastoma to cisplatin. Finally, radiation is the most effective therapy for medulloblastoma and is known to activate NFκB. Yet inhibition of NFκB with curcumin or other pharmacological and molecular inhibitors increases radiosensitivity in glioblastoma [54,55] and fibrosarcoma cells [49]. Increasing medulloblastoma sensitivity to radiation, especially if normal neuron sensitivity is unchanged, could dramatically change current treatment and improve outcomes for patients with medulloblastoma.

## Conclusions

We conclude that NFκB signaling may turn out to be extensively involved in medulloblastoma. Further successful treatment of medulloblastoma may rely on proper management of the NFκB pathway to ensure efficacy of current therapies. Much more work needs to be done to understand whether or not therapeutic manipulation of NFκB cellular regulation can be useful in a clinical setting.

## Competing interests

The authors declare that they have no competing interests.

## Authors' contributions

SES helped to conceive of the study, participated in study design and coordination, prepared the manuscript, performed annexin V staining, xenograft studies, and some dose-response testing. NJL carried out the cloning studies, subcellular fractionation, participated in study design, neurosphere experiments, dose-response testing and Western analysis. LAD participated in immunostaining, and Western analysis. HS helped to conceive of the study, and participated in its design and coordination. All authors read and approved the final manuscript.

## Pre-publication history

The pre-publication history for this paper can be accessed here:

http://www.biomedcentral.com/1471-2407/11/136/prepub

## Supplementary Material

Additional File 1 **Supplemental figure**. A - Construct for Tet-off dnIκB used in D425 cells to create 4H10 cells B - Neurospheres cultured in medium containing PDTC for 24 hours (photos at 10X) C - Transient transfection with p5X NFκB -Luc demonstrates diminished NFκB induction with dnIκB construct activated in 4H10 cells by removing DOX. Click here for file
