# Supplementary figures and images for: Inhibition of nuclear factor kappa-B signaling reduces growth in medulloblastoma in vivo
Source: BMC Cancer. 2011 Apr 14;11:136. doi: 10.1186/1471-2407-11-136 (PMC3094324; doi:10.1186/1471-2407-11-136)

## Slide 1
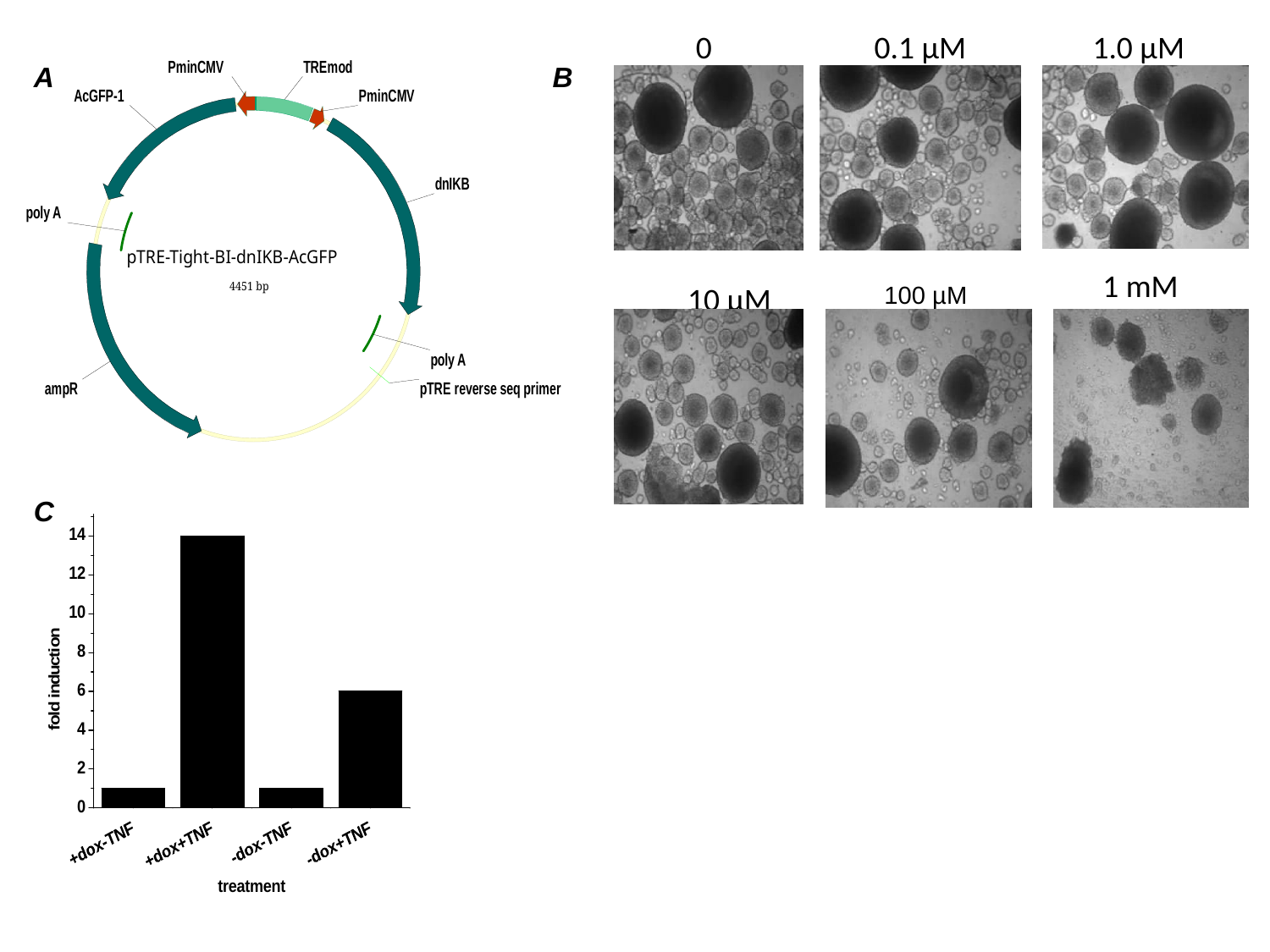

0
1 mM
100 µM
10 µM
0.1 µM
1.0 µM
A
B
C

Supplement: Additional File 1 — Supplemental figure. A - Construct for Tet-off dnIκB used in D425 cells to create 4H10 cells B - Neurospheres cultured in medium containing PDTC for 24 hours (photos at 10X) C - Transient transfection with p5X NFκB -Luc demonstrates diminished NFκB induction with dnIκB construct activated in 4H10 cells by removing DOX. [file 1471-2407-11-136-S1.PPT]
